# The PANGAEA study design – a prospective, multicenter, non-interventional, long-term study on fingolimod for the treatment of multiple sclerosis in daily practice

**DOI:** 10.1186/s12883-015-0342-0

**Published:** 2015-06-18

**Authors:** Tjalf Ziemssen, Raimar Kern, Christian Cornelissen

**Affiliations:** Zentrum für klinische Neurowissenschaften, Klinik und Poliklinik für Neurologie, Universitätsklinikum Carl Gustav Carus Dresden, Technische Universität Dresden, Fetscherstr. 43, D-01307 Dresden, Germany; Novartis Pharma GmbH, Roonstr. 25, D-90429 Nuernberg, Germany

**Keywords:** Multiple sclerosis, Relapsing remitting, RRMS, Fingolimod, Gilenya, Efficacy, Safety, Pharmacoeconomics, PANGAEA

## Abstract

**Background:**

Fingolimod (Gilenya®) is an oral medication for patients with highly active relapsing-remitting Multiple Sclerosis (RRMS). Clinical trials and post-marketing experience on more than 114,000 patients have established a detailed safety profile. Total patient exposure now exceeds 195,000 patient-years as stated in the last financial report (Dec 2014) of the Novartis Pharma AG, Basel, Switzerland. However, less is known about the safety of long-term fingolimod use in daily practice. Here, we describe the study design of PANGAEA (Post-Authorization Non-interventional German sAfety of GilEnyA in RRMS patients), a prospective, multicenter, non-interventional, long-term study to collect safety, efficacy, and pharmacoeconomic data on RRMS patients treated with fingolimod (0.5 mg/daily) under real-world conditions in Germany.

**Methods:**

PANGAEA is striving to assess a real-world safety and efficacy profile of fingolimod, based on data from 4,000 RRMS patients, obtained during a 60-month observational phase. A pharmacoeconomic sub-study of 800 RRMS patients further collects patient-reported outcome measures of disability, quality of life, compliance, treatment satisfaction, and usage of resources during a 24-month observational phase. Descriptive statistical analyses of the safety set as well as of stratified subgroups such as patients with concomitant diabetes mellitus and pretreated patients (e.g., natalizumab) will be conducted.

**Discussion:**

PANGAEA seeks to confirm the current safety profile of fingolimod obtained in phase I-III clinical trials. The study design presented here will additionally provide guidance on the therapeutic use of fingolimod in clinical practice and possibly assists physicians in making evidence-based decisions.

## Background

In Europe, fingolimod (Gilenya®) has been approved as an oral disease-modifying therapy (DMT) both for patients who have a highly active relapsing-remitting MS (RRMS) despite previous treatment with at least one DMT, and for patients who have a rapidly evolving severe RRMS [1].

Fingolimod is the prodrug of a sphingosine 1-phosphate (S1P) receptor agonist [2] that undergoes phosphorylation by sphingosine kinase in vivo [3]. Phosphorylated fingolimod interacts with S1P receptors expressed on the surface of immune cells, neurons, and cells of the cardiovascular system, leading to subsequent receptor internalization from the cell membrane. Fingolimod then induces polyubiquitination and degradation of internalized S1P receptors, thereby preventing immune cells expressing S1P receptors from sensing the S1P gradient between lymph-nodes and periphery and, hence, from exiting the lymphoid tissue [4]. This prevention consequently results in a long-term functional antagonism that differentially affects subsets of lymph-node immune cells and *per se* attenuates autoimmune pathology [5, 6]. In MS, the mechanism of action of fingolimod prevents infiltration of autoreactive lymphocytes into the central nervous system. Preclinical data further suggest that fingolimod has also direct effects in the central nervous system, reducing demyelination and promoting remyelination [7].

In 2010, two phase-III studies, FREEDOMS [8] and TRANSFORMS [9], demonstrated the efficacy of fingolimod in patients with RRMS. FREEDOMS, a placebo-controlled trial, showed a relative reduction of the relapse rate by 54 % to 60 % with corresponding effects on disability progression and magnetic resonance imaging (MRI)-related measures [8]. These beneficial effects on relapse rate were reproduced by FREEDOMS II [10]. TRANSFORMS then demonstrated superiority of fingolimod over treatment with intramuscular interferon beta-1a, with significantly lower relapse rates and better MRI outcomes [9].

Adverse events reported for patients treated with fingolimod might be caused by its interaction with S1P receptors both inside and outside the immune system. The first dose of fingolimod needs to be applied under medical supervision because heart rate and atrioventricular conduction time might decrease within the first hours after the first application of fingolimod [11, 12]. The risk of certain infections might be increased by a dose-dependent but reversible reduction of blood-lymphocyte counts by fingolimod [13], but the overall rate of infections under fingolimod is similar to placebo [8]. However, since two fatal cases of varicella-zoster virus infection were reported in fingolimod-treated patients, [9], varicella-zoster virus immune status has to be assessed before fingolimod treatment initiation. In certain subsets of patients, such as patients with diabetes mellitus or a history of ophthalmological abnormalities, fingolimod has been further associated with macular edema that occurred in 0.4 % of fingolimod-treated patients and usually resolves after discontinuation of medication [8, 9, 14].

The experience both in clinical trials and in the post-marketing setting now exceeds 114,000 fingolimod-treated patients or 195,000 patient-years on fingolimod [15]. This experience has established a well-characterized safety and efficacy profile of fingolimod. However, validity for the real-world treatment setting might be considered being different to the situation in clinical trials due to the number of patients enrolled and selection criteria applied [11, 16]. We therefore sought to obtain a more generalizable safety and efficacy profile on the basis of long-term experience with fingolimod in daily routine practice. To address the above mentioned and other safety cenocerns, a detailed data acquisition and monitoring algorithm taking the Risk Management Plan (RMP) of the European Medicines Agency (EMA) into consideration was established within the study design, also to support physicians in implementing the RMP in daily clinical practice.

Here we report the study design of the prospective, multicenter, non-interventional, long-term study PANGAEA ((Post-Authorization Non-interventional German sAfety of GilEnyA in RRMS patients). PANGAEA will provide data of German RRMS patients treated with fingolimod under real-life conditions over a period of 5 years. The primary aim of this study is to confirm the safety and efficacy profile of fingolimod obtained in phase I-III clinical trials in real-world conditions. In addition, pharmacoeconomic data will be collected from a subset of patients.

## Methods/Design

### Study design

**Fig. 1:**
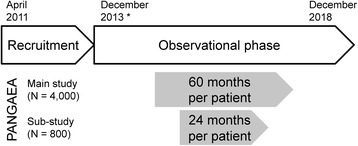
Timeline of the PANGAEA main study and pharmacoeconomic sub-study. Main study and sub-study will include 4,000 and 800 RRMS patients treated with fingolimod (0.5 mg/daily), and the observational phase will be 60 months and 24 months, respectively (* in the pharmacoeconomic sub-study, recruitment will end after 800 patients)

PANGAEA is a prospective, multicenter, non-interventional, long-term study in RRMS patients treated by fingolimod (0.5 mg daily) in routine practice. To confirm the safety and efficacy profile of fingolimod in real-world conditions, data from approx. 4,000 RRMS patients from approx. 500 neurological centers and practices in Germany were included into PANGAEA. The number of participants per practice or center ranges from 1 to 50 and more. In addition, a pharmacoeconomic sub-study with 800 RRMS patients treated in approx. 180 neurological clinics and practices will systematically collect patient-reported measures on disability, quality of life (QoL), compliance, treatment satisfaction, and consumption of resources. Centers experienced in phase IV observational trials were asked to participate in this sub-study. Selection criteria included participation in former Novartis sponsored observational trials, experienced in the documentation of patient reported outcome measurements, center size, and scientific interest in the sub-study. The recruitment period of the PANGAEA main study and sub-study started in April 2011. In the PANGAEA main, data are obtained during a 60-month observational phase per patient and observations will be completed on December 2018 (Fig. 1). The documentation of the sub-study data ends after 24 months. Patients participating in the sub-study will continue the main-study documentation until month 60. This observational study was sponsored and executed by the Novartis Pharma GmbH, Nuremberg, Germany.

### Study population

Participants are eligible if they were diagnosed with RRMS [17], if their physicians decide to prescribe fingolimod independently of participation in the study, and after informed consent has been provided. All fingolimod receiving patients are included into PANGAEA. This includes patients that receive fingolimod for the first time in PANGAEA (main- and sub-study) and patients that are already treated with fingolimod and started the therapy within a former clinical trial (only into the main study). There are no exclusion criteria except for the contraindications mentioned in the respective summary of the product information [18].

The prevalence of MS in Germany is estimated to be 150 cases per 100,000 inhabitants, which is equivalent to about 122,000 MS patients [19]. Assuming an enrollment of 5 patients per practice or 4,000 patients in total, the enrolled number of study participants as well as the duration of the observational study period of 60 months is deemed sufficient to detect the most commonly occurring adverse events (AEs). Sample size of the pharmacoeconomic sub-study was limited to 180 MS-centers or 800 MS patients.

### Procedures

According to routine practice and as recommended by the German Society of Neurology [17], in the main-study visits take place every 3 months for a period of 60 months, once the first month of treatment is over (first visit). Data for pharmacoeconomic sub-study are documented every 3 months for a period of 24 months per patient.

**Fig. 2:**
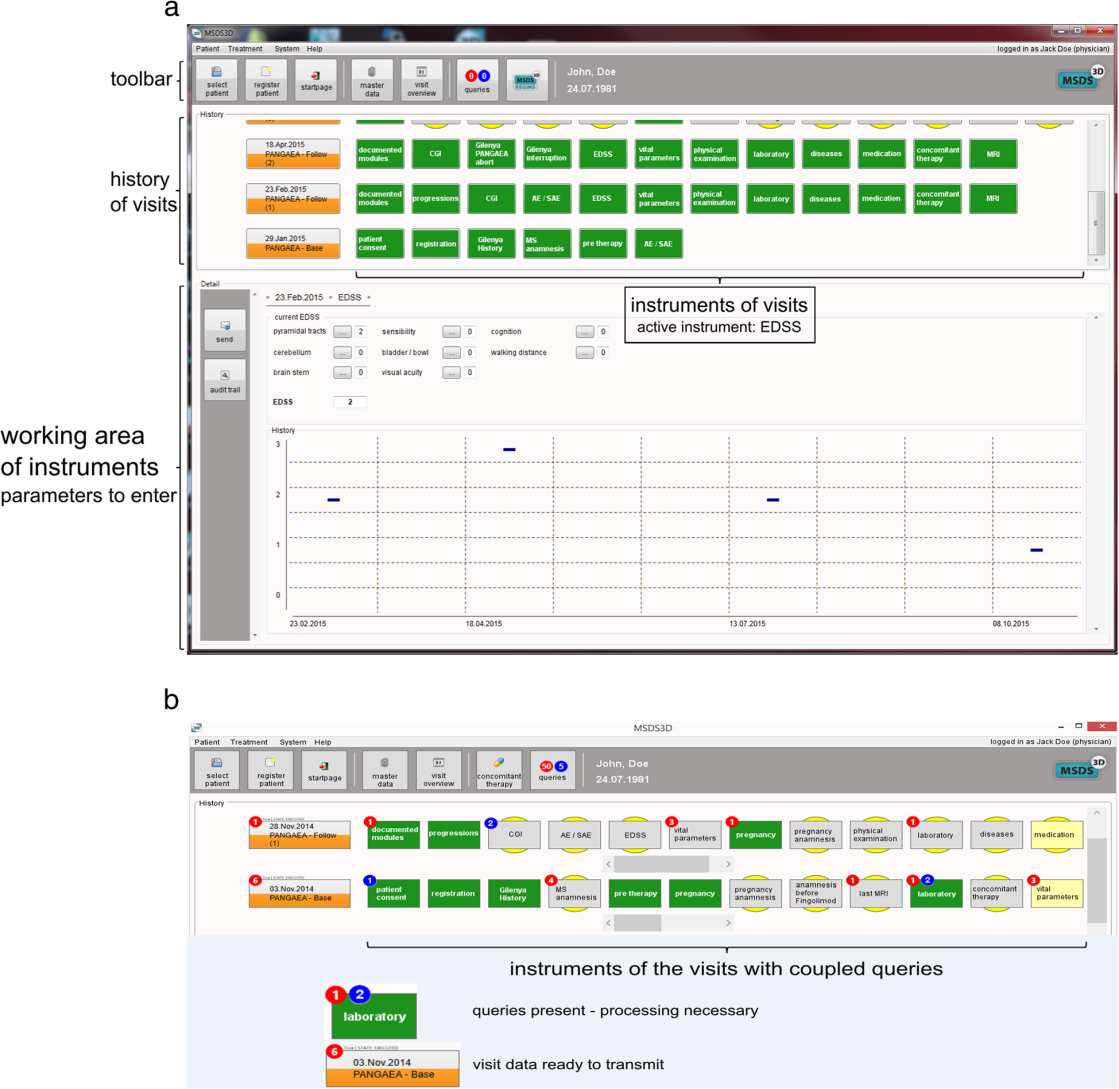
MSDS 3D PANGAEA module. Baseline and follow up visits are horizontally presented with boxes representing examinations. The lower part of the screen exemplary shows the EDSS data entry menu (**a**). **b** depicts in detail the horizontal presentation of completed and uncompleted visits and examinations (denoted by colors as indicated)

Demographic and clinical data of participants are obtained from interviews or medical examinations and are collected by the treating neurologist. Questionnaires on patient-reported outcomes are completed by participants at regular visits in the presence of a health professional. All data are collected using standardized electronic case report forms. Data are entered online at study sites, using either a customized web-based data entry tool or the software-based MS management system 3D (MSDS 3D; [20]). The PANGAEA MSDS 3D module further guides neurologists and MS-nurses through treatment management, including first dose monitoring, ophthalmological examinations, and regular laboratory follow ups. On the software interface, all procedures are displayed in clickable boxes leading to menus for data entry. Upon authorization, data might be entered by the neurologist (e.g., EDSS, adverse effects) or the MS-nurse (e.g., questionnaires). From the MSDS 3D start screen, details of upcoming, past, and missed appointments can be directly assessed (Fig. 2a,b). Anonymity and data protection are ensured by a complex process including encrypted transfer.

At the time of entry, data quality is ensured by validation checks. Data are daily reviewed by the database coordinator. Data management is overseen by the data management team of the Clinical Research Organization responsible (Kantar Health GmbH).

### Measures main study

#### Baseline assessments

All measurements are summarized in Table 1. At baseline, patient histories are documented in addition to the patient informed consent and demographic patient characteristics. Among others, data on the patient’s history include the time since first symptoms and diagnosis of MS, the number of lesions in T2-weighted MRI and gadolinium-enhancing (Gd^+^) lesions, and the number of relapses within 12 and 24 months before study start.

**Table 1:** Data to be obtained during the PANGAEA main study

	Baseline	Month 1	Month 3	Every 3 months	Final visit (60 months)
Baseline Assessments					
Informed consent	X				
Patient characteristics	X				
Anamnesis (incl. MS)	X				
First dose observation					
12-channel ECG	X	– additionally before first dose and when restarting fingolimod after treatment interruption –			
HR, BP, bradycardia	X				
Precautions for treatment					
Varicella-zoster status	X				
Prev. immunotherapy	X				
Concomitant diseases and drugs	X				
Impaired lung function	X				
Chronic infections	X				
HR, BP	X	X	X	X	X
CBC, clin. chemistry	X	X	X	X	X
Ophthalmology	– if required –		X	– if required –	
Pregnancy	X	– if required –			
Prem. discontinuation			X	– if required –	
Monitoring of disease progression					
CGI	X	X	X	X	X
EDSS	X	X	X	X	X
MSFC^a^	X	X	X	X	X
SDMT^a^	– every 6 months –				
MS-relapse		X	X	X	X
MRI-lesions		– if available –			
Adverse Events					
AE		X	X	X	X
SAE		X	X	X	X

*HR*: heart rate, *BP*: blood pressure, *CBC*: complete blood count, *CGI*: Clinical Global Impression scale, *EDSS*: Kurtzke’s Expanded Disability Status Scale, *MSFC*: Multiple Sclerosis Functional Composite, *SDMT*: Symbol Digit Modalities Test, *MRI*: Magnetic Resonance Imaging, *AE*: Adverse events, *SAE*: Serious Adverse Events

^a^MSFC and SDMT are assessed in a subset of practices and centers

As PANGAEA documents daily clinical practice, MRI data were asked to be documented by the neurologist of the center if a MRI was performed. Therefore MRI acquisition was not part of the study protocol and was not performed via standardized protocols. MRI read outs were not evaluated via a central reading facility.

#### First dose observation

The first dose of fingolimod is applied under clinical observation because of the potential risk of bradycardia and decreased atrioventricular conduction. In detail, at baseline as well as 6 h after the first dose of fingolimod, a 12-channel ECG is performed to identify abnormalities. Furthermore, heart rate, blood pressure, and the occurrence of symptoms that might indicate bradycardia are examined at 1 h intervals during the 6 h post-dose period. A continuous monitoring of heart function by long-term (Holter) ECG is recommended during this period. If heart rate is lowest at the end of the post-dose observation, monitoring is extended for at least two hours. If patients develop clinically significant symptoms indicating bradycardia or atrioventricular block, clinical management should be initiated as required, and monitoring is continued at least overnight and until the symptoms resolve. This first-dose monitoring should additionally be performed if patients required pharmacological intervention during first-dose monitoring (second-dose monitoring), and, importantly, if fingolimod therapy has been interrupted.

#### Precautions for treatment

Varicella zoster status, previous immunomodulatory treatments, concomitant drugs and concomitant diseases such as diabetes mellitus, impaired lung function, and chronic infections are documented at baseline. Other safety precautions include the assessment of blood pressure, heart rate, complete blood count (lymphocyte count), and clinical chemistry such as liver enzymes (transaminases) and blood lipids (triglycerides, high-density and low-density lipoprotein [HDL, LDL]) at every visit beginning at baseline. Ophthalmological examinations are performed after 3 months or at any visit if required. Female patients are tested for pregnancy at baseline and, if required, at follow-up visits. Premature discontinuation of therapy is documented at any study visit beginning at month 3.

#### Monitoring of disease progression

Global symptomatology and treatment response are assessed by the Clinical Global Impression scale (CGI; [21]) at every visit beginning at baseline. Disability is scored by means of the Kurtzke’s Expanded Disability Status Scale (EDSS) [22]. MS-relapses and number of MRI lesions, where available, are documented at every visit beginning at month 1. In some centers, additional data on MS symptomatology and cognitive processes most frequently affected by MS are obtained by the Multiple Sclerosis Functional Composite (MSFC [23], every visit beginning at baseline) and the Symbol Digit Modalities Test, respectively (SDMT [24], every 6 months).

#### Adverse events

At every visit, the investigators evaluate the occurrence of AEs and serious AEs. AEs are defined as any unfavorable change in the patients’ pretreatment condition, regardless of their potential relation to treatment and irrespective of whether medication was taken as intended. Each AE is to be characterized by type. The time of first occurrence, duration, and intensity of an AE, as well as the causal relationship to therapy, counteractive measures and outcomes of AEs are to be documented. Serious AEs comprise lethal or life threatening events, hospitalizations, events leading to major incapacity, persistent or significant disability or incapacity, congenital anomaly or birth, and events that are otherwise medically significant. The latter may also apply to abnormal laboratory values and test results.

### Measures sub-study

Measurements of the pharmacoeconomic sub-study comprise patient-reported disability, QoL, treatment compliance, treatment satisfaction, and consumption of resources (Table 2). At baseline and after 12 and 24 months, the patients’ perceived disability is assessed by the UK (Guy’s) Neurological Disability Scale (UKNDS; [25]). At baseline and every 6 months, patient-reported QoL is evaluated by both the standardized EuroQol (EQ)-5D questionnaire [26], and the QoL- and activity subscale of the Patient Reported Outcome Indices for Multiple Sclerosis (PRIMUS; [27]).

**Table 2:** Data to be obtained during the PANGAEA pharmacoeconomic sub-study

	Baseline	Follow up visits	Final visit (24 months)
UKNDS	X	at 12 months	X
EQ-5D, PRIMUS-A, PRIMUS-L	X	every 6 months	X
Compliance questionnaire	X	every 3 months	X
TSQM-9	X	every 3 months	X
Consumption of resources	X	every 3 months	X

*UKNDS*: UK (Guy’s) Neurological Disability Scale; *EQ-5D*: Euro quality of life questionnaire, *PRIMUS*: Patient Reported Outcome Indices for Multiple Sclerosis (subscale *A*: activity, *L*: quality of life); *TSQM-9*: Treatment Satisfaction Questionnaire for Medication

Data on the patient-reported compliance with therapy are obtained through the use of a compliance questionnaire at baseline and every 3 months. The compliance questionnaire consists of five yes/no questions asking whether and when treatment was eventually discontinued; one free text field asks for the number of days the MS-medication was not taken. Treatment satisfaction is evaluated by the Treatment Satisfaction Questionnaire for Medication (TSQM-9; [28]) at baseline and every 3 months. In this questionnaire, patients are asked to rate their satisfaction with nine different aspects of MS-treatment on a 7-point Likert scale ranging from ‘1’ (very dissatisfied) to ‘7’ (very satisfied).

The questionnaire on patient-reported consumption of resources is completed at baseline and every 3 months. Several multiple-choice questions ask for information on demographic details, on employment, and on health and long-term-care insurances. Yes/no-questions combined with free text fields ask patients about their expenditures on medication, treatments, and devices, the type of outpatient treatment and specialized medical consultations, their participation in patient and education programs, and their responsibility for relatives. There are additional free text fields in which patients are asked to specify the type and extent of inpatient and outpatient care caused by MS-relapses. In one question, patients are asked to assess their work productivity on a 10-point Likert scale with ‘0’ corresponding to being ‘not affected’ and ‘10’ corresponding to being ‘completely affected by MS’.

### Statistical analysis

All analyses are based on the safety population defined as all included patients who received at least one dose of fingolimod. Patients are excluded if no follow-up information is available. Missing data are not replaced. Continuous data are described as mean ± standard deviation (SD), minimum, median, maximum, 5th percentile, 1st and 3rd quartile, 95th percentile, and number of non-missing values. Nominal- and ordinal-level data are reported in terms of absolute and relative frequencies. The incidence rates with 95 %-confidence intervals are determined for all safety outcomes. Incidence rates for pre-specified subgroups such as patients with concomitant diabetes mellitus and pretreated patients will additionally be evaluated (e.g., interferons, glatirameracetat, mitoxantrone, azathioprine, natalizumab). For all analyses, the statistical software program SAS® Version 9.2 (and above) is used.

### Ethical considerations

The steering committee consisting of neurologists, internists, and pharmaco-epidemiologists advises on study design and data analysis, and an independent data monitoring committee is responsible for review of the ongoing safety of patients enrolled in the study. Regionally competent ethics committees are consulted in accordance with both the codex of the Voluntary Self-Regulation of the Pharmaceutical Industry (FSA; [29]) and recommendations dealing with quality aspects of non-interventional observational studies [30, 31]. The study is registered at CFTY720DDE02 [32].

## Discussion

Here we report on the study design of the prospective, multicenter, non-interventional, long-term study PANGAEA in fingolimod-treated RRMS patients. This large, methodologically precise study evaluates safety-relevant data of fingolimod treatment under routine practice conditions in Germany over a period of 5 years. In addition, long-term efficacy data as well as pharmacoeconomic data will be collected. Although there is growing clinical experience with fingolimod, data on the long-term use of the medication in daily routine are currently still limited. PANGAEA has therefore been designed to provide definitive, long-term data on patient- and treatment-related parameters that have been identified as safety relevant in clinical trials and post-marketing experience.

To manage the large amounts of data that accumulate during long-term treatment, the software-based management system MSDS 3D is employed in some centers and practices participating in PANGAEA. MSDS 3D was developed in 2010 to interactively collect patient data, to facilitate analysis and interpretation, and to assist neurologist in executing complex processes required for MS diagnosis, treatment initiation, and long-term therapy [33]. Due to the modular structure of MSDS 3D, neurologists are guided through all necessary medical investigations [20]. Importantly, both the MSDS 3D-software and the PANGAEA study design also provides guidance on the therapeutic use of fingolimod in clinical practice, starting with preparatory examinations, first-dose application, and long-term treatment. Since the structure and content of data acquisition described in this design reflects the RMP, the treatment and monitoring algorithm provided will ensure the best possible safety for RRMS patients treated with fingolimod in the long term.

Due to exclusion criteria subjects of clinical MS trials might represent a subgroup of patients with less concomitant diseases in comparison to the general MS population that might suffer from concurrent conditions such as hypertension, coronary artery, diabetes mellitus, and ocular disease. The study design of PANGAEA, along with both the proposed high number of patients and the long study duration, will therefore enable us to assess efficacy and safety parameters in certain subgroups of patients, for example those with concomitant diabetes mellitus.

In patients with diabetes mellitus, fingolimod treatment might cause a higher risk of macular edema. In renal transplant studies, the rate of macular edema after fingolimod treatment was higher in patients with diabetes mellitus than in patients without [14]. However, in these studies, fingolimod doses were up to 10-fold higher than the dose approved for MS. Since RRMS patients with diabetes mellitus were excluded from phase-III clinical trials, the incidence of macular edema in fingolimod-treated (0.5 mg) RRMS patients with diabetes is currently unknown and needs clarification. Until now, only case reports have been published on fingolimod treated RRMS patients with concomitant diseases.

PANGAEA will further analyze the effects of concomitant and prior medications on efficacy and safety issues with fingolimod, thereby considerably strengthen its safety profile. One post-marketing case report, for example, documented worsening MS under fingolimod given after a period of natalizumab pretreatment [34]. However, no causal relationship could be established, and a recent observational study in France [35] demonstrated that the occurrence of relapses during the natalizumab washout period is the only prognostic factor for MS relapses after fingolimod initiation. The authors therefore recommended limiting the washout period to less than 3 months. PANGAEA will additionally allow the analysis of and recommendation for subgroups of MS patients as defined by other previous immunomodulatory therapies such as interferons, glatirames acetate, mitoxantrone, and azathioprine.

As the first oral DMT approved for the treatment of MS [36], fingolimod may increase treatment adherence over that observed with parenteral therapies. Although patients who struggle with adherence to injectable medications because of needle phobia, injection-site reactions, and side effects might decide to initiate fingolimod, clinicians need to be aware of the developing safety profile of fingolimod. Therefore, the long-term data being collected during PANGAEA will allow a significantly broadened evaluation of the well described safety profile of Fingolimod established from phase I-III clinical trials and will allow physicians to make informed, evidence-based decisions regarding its use in daily practice.
